# Frontal Theta Dynamics during Response Conflict in Long-Term Mindfulness Meditators

**DOI:** 10.3389/fnhum.2017.00299

**Published:** 2017-06-07

**Authors:** Han-Gue Jo, Peter Malinowski, Stefan Schmidt

**Affiliations:** ^1^Department of Psychiatry, Psychotherapy and Psychosomatics, Medical Faculty, RWTH Aachen UniversityAachen, Germany; ^2^Department of Psychosomatic Medicine and Psychotherapy, Medical Faculty, Medical Center—University of FreiburgFreiburg, Germany; ^3^Research Centre for Brain and Behaviour, Liverpool John Moores UniversityLiverpool, United Kingdom

**Keywords:** theta activity, phase synchrony, cognitive control, response conflict, meditation

## Abstract

Mindfulness meditators often show greater efficiency in resolving response conflicts than non-meditators. However, the neural mechanisms underlying the improved behavioral efficiency are unclear. Here, we investigated frontal theta dynamics—a neural mechanism involved in cognitive control processes—in long-term mindfulness meditators. The dynamics of EEG theta oscillations (4–8 Hz) recorded over the medial frontal cortex (MFC) were examined in terms of their power (MFC theta power) and their functional connectivity with other brain areas (the MFC-centered theta network). Using a flanker-type paradigm, EEG data were obtained from 22 long-term mindfulness meditators and compared to those from 23 matched controls without meditation experience. Meditators showed more efficient cognitive control after conflicts, evidenced by fewer error responses irrespective of response timing. Furthermore, meditators exhibited enhanced conflict modulations of the MFC-centered theta network shortly before the response, in particular for the functional connection between the MFC and the motor cortex. In contrast, MFC theta power was comparable between groups. These results suggest that the higher behavioral efficiency after conflicts in mindfulness meditators could be a function of increased engagement to control the motor system in association with the MFC-centered theta network.

## Introduction

To optimize behavior, the brain is thought to monitor the presence of competing information and resolve the conflict. This cognitive process has been studied with various cognitive tasks, such as the Stroop, the Simon and the Flanker tasks, in which interference during the response selection stage causes response conflict. Converging evidence suggests that the medial frontal cortex (MFC), which included the anterior cingulate cortex (ACC), plays a significant role in the processing of response conflicts (Botvinick et al., 2004; Ridderinkhof et al., 2004). Recent theories of MFC functions postulate that the MFC is involved in detecting interference of competing actions and interacts with other brain areas in order to overcome the conflict and to achieve goal-directed behavior. Accordingly, over the past decade, frontal neural oscillations in the theta-band (4–8 Hz) recorded over the MFC have been linked to conflict processing (for a review see Cavanagh and Frank, 2014). Evidence indicates that when conflicts are present, theta power over the MFC is increased. Usually, this response is not phase-locked to the stimulus onset (Cohen and Cavanagh, 2011; Nigbur et al., 2011, 2012; Cavanagh et al., 2012) and may serve as a neural marker that predicts the timing of the upcoming response. In addition to theta power increases, theta phase synchrony between distant brain areas is increased after conflicts. This is understood as a functional mechanism for the integration and exchange of information between brain areas. Theta phase synchronization studies provided evidence that the MFC recruits the dorsal lateral prefrontal cortex (DLPFC), the motor cortex, as well as the right parietal cortex, to implement motor responses according to goal-directed values (Cavanagh et al., 2009; van de Vijver et al., 2011; Nigbur et al., 2012; van Driel et al., 2012). Such MFC-centered theta networks were observed in cases where behavioral adjustments were required, suggesting a direct involvement in conflict resolution and the control of the motor system to avoid errors.

**Figure 1:**
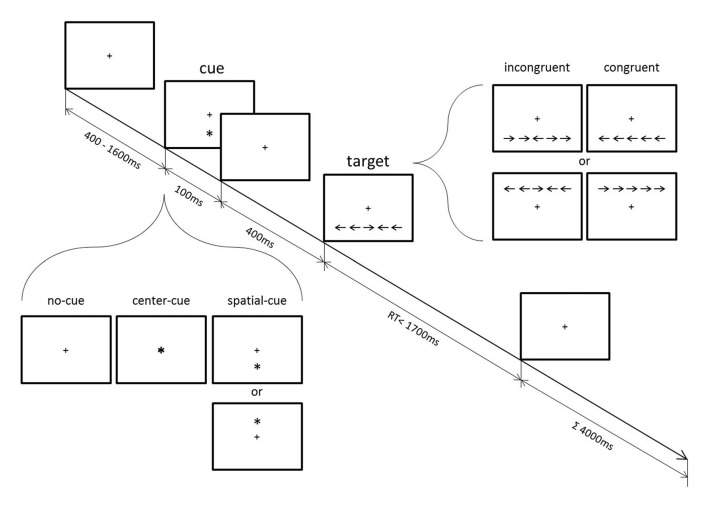
Schematic diagram of the attention network test (ANT; modified from Jo et al., 2016).

Furthermore, greater efficiency in controlling response conflicts has been repeatedly reported among individuals who have practiced mindfulness meditation (e.g., Jha et al., 2007; Tang et al., 2007; Moore and Malinowski, 2009). Mindfulness meditation is a specific self-regulation technique, which aims at achieving a mental state of non-judgmental awareness in the present moment. Therefore, it is expected that individuals who regularly engage in meditation practices that include mindfulness techniques would increase the awareness of their motor intention (Jo et al., 2014, 2015) and show enhanced skills in controlling motor responses. For instance, recent studies on conflict effects using the flanker-type attention network test (ANT, Fan et al., 2002; see Figure 1) showed fewer error responses among meditators than non-meditators (van den Hurk et al., 2010; Jo et al., 2016). This was particularly the case for incongruent trials, in which interference is created during the response selection stage. It is notable that reaction times (RTs) were comparable between groups, indicating a more efficient use of resources in meditators rather than a speed-accuracy tradeoff. However, although the positive effect of meditation techniques on cognitive control is robust, the neural mechanisms underlying the greater efficiency in response conflicts are unclear.

Previous fMRI studies have shown that functional changes in the MFC and ACC are linked to the effects of mindfulness meditation. Greater activation of the MFC and ACC was seen during mindfulness of breathing in experienced meditators (Hölzel et al., 2007). A longitudinal study also showed increased activation of these brain areas during resting state after 5 days of integrative body-mind training (IBMT; Tang et al., 2009). Another brain region that showed functional changes after 6 weeks of mindfulness training is the DLPFC, where the trained group showed greater activation during response conflict (Allen et al., 2012). Since these brain regions have been implicated in response conflict processing in association with MFC theta dynamics, it is assumed that greater behavioral efficiency among meditators may be reflected in the functional differences in frontal theta dynamics. Indeed, EEG research has demonstrated that meditation training modulates theta activity over frontal areas involved in cognitive processing and attention. Increased local theta synchrony over the frontal electrodes corresponded to target perception, in particular for those individuals who participated in intensive meditation training (Slagter et al., 2009). Changes in frontal theta power, as well as theta synchrony between distant brain areas, were also seen in long-term meditators during internally guided states of meditation (Aftanas and Golocheikine, 2001). Moreover, a recent study with expert meditation practitioners suggests that frontal theta activity is associated with reduced susceptibility to mind wandering (Brandmeyer and Delorme, 2016). In line with these findings, frontal slow oscillations in the delta and alpha ranges during meditation have also been proposed to be linked with maintaining top-down control of attention (Harmony, 2013; Lomas et al., 2015).

**Figure 2:**
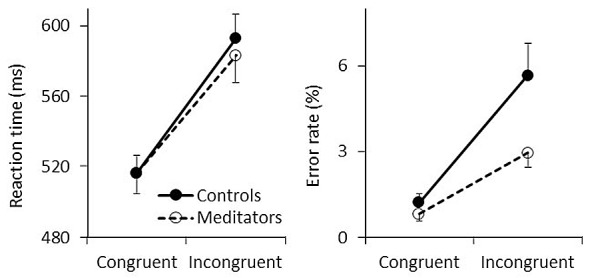
Behavioral results. Mean reaction time (RTs; left) and error rates (ERs; right) are shown for congruent and incongruent trials. Solid and dashed lines represent controls and meditators, respectively. Error bars represent standard errors of the means.

The aim of the present study was to investigate frontal theta dynamics in mindfulness meditators. We examined whether mindfulness meditators who have shown greater efficiency in a response conflict task (Jo et al., 2016; Figure 2) would also display differences in MFC theta power and/or MFC-centered theta network compared to a control group in trials where they showed increased response accuracy after conflicts. Based on previous indications that the MFC-centered theta network is more relevant for error-related processing, we assumed that behavioral efficiency (indexed by fewer error responses) is primarily associated with the MFC-centered theta network rather than MFC theta power.

## Materials and Methods

### Participants and Task Design

Twenty-five long-term mindfulness meditators (15 females; mean age 40.6 years, SD = 8.64) were recruited through mailing lists and flyers distributed at various meditation centers. Inclusion criteria were at least 5 years of meditation practice, with a practice frequency of at least three times per week during the last 3 months. On average, meditators had 13.1 years (SD = 5.9) of meditation experience and meditated 247.8 min (SD = 104.9) per week. Twenty-five healthy matched controls of the same gender and age (15 females; mean age 40.4 years, SD = 8.80) were recruited through advertisements in the University Medical Center of Freiburg. Participants in the control group had no prior experience of contemplative practice including meditation, Tai Chi, Qi Gong and Yoga. The maximum age was set at 50 years for all participants. Further exclusion criteria were a history of psychiatric conditions, neurological diseases and visual impairment that cannot be corrected by means of visual aid.

One participant of each group who had a high ratio of incorrect responses (greater than three standard deviations from the group mean) and three participants (two meditators and one control), who had high EEG artifacts (see below), were excluded from the analysis. Thus, we compared 22 long-term mindfulness meditators (see Table 1 for the characteristics of the meditation experience) with 23 healthy matched controls. The achieved power (1-β) of the present matched-pairs design (total sample size = 45) is 0.75 when the effect size and α level are 0.4 and 0.05 (two-tailed), respectively.

**Table 1:** The characteristics of the meditation experience of the meditating group.

	Meditators
**Tradition**
Karma Kagyü (Tibetan)	2
Vipassana	6
Soto—Zen	1
Mantrameditation	3
Mindfulness (Kabat-Zinn)	1
Osho Kundalini Meditation	1
Not specified	8
**Experience (frequency)**
7 times/week	17
5–6 times/week	2
2–4 times/week	3
Minutes/week	243.9 (minutes)

Most of the participants reported that they were right-handed except for three meditators (two left-handed and one mixed-handed) and four controls (one left-handed and three mixed-handed). Three controls and one meditator did not report their handedness. This study was approved by the ethics committee of the University Medical Center of Freiburg. All participants gave written informed consent in accordance with the Declaration of Helsinki. As listed in Table 1 the long-term meditators were engaged in a range of different meditation traditions. It is crucial to take such differences into account in studies on the effects of *specific* meditation practices. However, as this study is concerned with generic mechanisms of cognitive control, which are thought to be a basic principle of all types of meditation practices that involve a mindfulness component (Malinowski, 2013), it was deemed appropriate to consider them together. Therefore, any effects found in this study should be considered to be relatively generic.

Participants performed the flanker-type ANT (Figure 1) with concurrent EEG recording. The ANT has been used to examine the efficiency of alerting, orienting and conflict functions independently from each other within a computerized single task that combines a cued detection and flanker-type paradigm. Response conflict was introduced by surrounding the left- or right-pointing target arrow with either congruent or incongruent flankers. Cues presented prior to the appearance of the target arrow provided modulations of alerting and orienting functions (see below).

Participants held a two-button computer mouse with both hands and each thumb was placed on one mouse button. A fixation cross at the center of the screen was always displayed. After a random period varying between 400 ms and 1600 ms, a cue (an asterisk) appeared for 100 ms in one of the following positions: above or below the fixation cross (spatial-cue), in the center (center-cue), or not at all (no-cue). Five-hundred milliseconds after the cue stimulus onset, a target arrow appeared for a maximum duration of 1700 ms either above or below the fixation cross. The target arrow was horizontally surrounded by two flanker arrows on each side, which pointed either in the same direction (←←←←← or →→→→→, congruent target) or in the opposite direction (→→←→→ or ←←→←←, incongruent target) as the central arrow. Participants were asked to press either their left or right thumb as fast and as accurately as possible depending on the direction of the central target arrow. The duration of each trial was 4000 ms. After a 24-trials practice, participants had to perform three blocks of trials. Each block comprised 96 pseudo-randomized trials that consisted of 48 congruent and 48 incongruent trials, one third of which were either no-cue, center-cue, or spatial-cue trials (32 each). Since the present study focused on response conflict effects, the trials were only analyzed according to congruency, which allow the estimation of an individual conflict effect irrespective of the particular cueing condition (Fan et al., 2002). Behavioral data and ERP analyses in respect of cueing conditions can be found in Jo et al. (2016). In brief, whereas cueing conditions revealed no group effect, compared to the control group, mindfulness meditators showed fewer error responses and exhibited higher parietal P3 amplitude during incongruent trials irrespective of response timing. The spectral data reported here have not been included in the previous study. Participants are not exactly the same as in the previous study due to exclusions based on EEG artifacts, but this difference is minor and does not affect the results.

### EEG Recordings and Pre-Processing

EEGs were recorded with a 64-channel DC-EEG amplifier using active electrodes (Brain Products, Germany) without applying low cut-off and notch filters. An initial reference was placed at FPz and sampling rate was set at 500 Hz. One channel EOG was recorded to monitor ocular movements. Using Matlab (Mathwork, Inc., Natick, MA, USA) and EEGLAB toolbox ver. 13 (Delorme and Makeig, 2004), EEG data were first down-sampled offline to 250 Hz and high-pass filtered at 0.1 Hz. After segmentation into epochs ranging from −2000 ms to 2000 ms from target stimulus onset, ocular artifact correction was performed using independent component analysis (ICA) with visual inspection of EOG recording. Trials containing EMG and other artifacts were manually removed. For further analyses only correct response trials with a maximum RT of 1000 ms were selected. On average, 95.6% trials (meditators, 95.8%; controls, 95.4%; *p* = 0.818) and 92.4% trials (meditators, 93.3%; controls, 92.4%; *p* = 0.375) were analyzed for congruent and incongruent conditions, respectively.

### Time-Frequency Analyses

All EEG segments were converted to current source density (CSD) using the method described by Kayser and Tenke (2006), which is based on the spherical spline surface Laplacian algorithm (Perrin et al., 1989). CSD transformation highlights the local electrical activities and diminishes the volume conduction effects. Although CSD is considered an estimate of electrical activity in neuronal populations, it should not be generalized to source-based activity. Time-frequency analysis from 2 Hz to 45 Hz were then computed using a Morlet wavelet implemented in EEGLAB with the number of cycles increasing linearly with frequency, from 2 cycles at the lowest frequency to 22.5 at the highest. Time-frequency decomposition was performed for target stimulus-locked and response-locked epochs. For the latter, the EEG segment was re-sorted to be time-locked to the response onset. Time-frequency decomposition produced instantaneous complex number *z(f,t)*, where *f* is the frequency and *t* is the time point at 125 Hz. From the resulting complex number, we estimated power as *p*(*f,t*) = *real[z(f,t)]*^2^ + *imag[z(f,t)]*^2^ for each frequency. In order to minimize sensitivity to noisy trials, each trial was normalized by dividing each frequency by the mean spectral power of the frequency over the full length of the segment for each participant (Grandchamp and Delorme, 2011). After computing the trial average for each condition and each frequency, baseline correction was applied by dividing the mean spectral power for the period ranging from −300 ms to −100 ms before the cue stimulus onset (i.e., from −800 ms to −600 ms before the target stimulus onset). This baseline correction was applied for both target stimulus-locked and response-locked epochs. The outcomes were then converted to a decibel scale (*10 × log10 [power/baseline]*).

Instantaneous phase angle was defined as φ(*f,t*) = *angle*[*z*(*f,t*)] and used to compute inter-channel phase synchronization (ICPS) between two channels. ICPS is defined as $N^{-1}\left|\sum e^{i\theta \;(f,t)}\right|$, where *N* is the number of trials and *θ* is the phase angle difference between two given electrodes, *φ*_1_(*f,t*) − *φ*_2_(*f,t*). ICPS ranges from 0 to 1 and values close to 1 indicate higher phase synchrony over trials between two electrodes.

### Selection of Frequency Bands, Electrodes and Time-Windows

**Figure 3:**
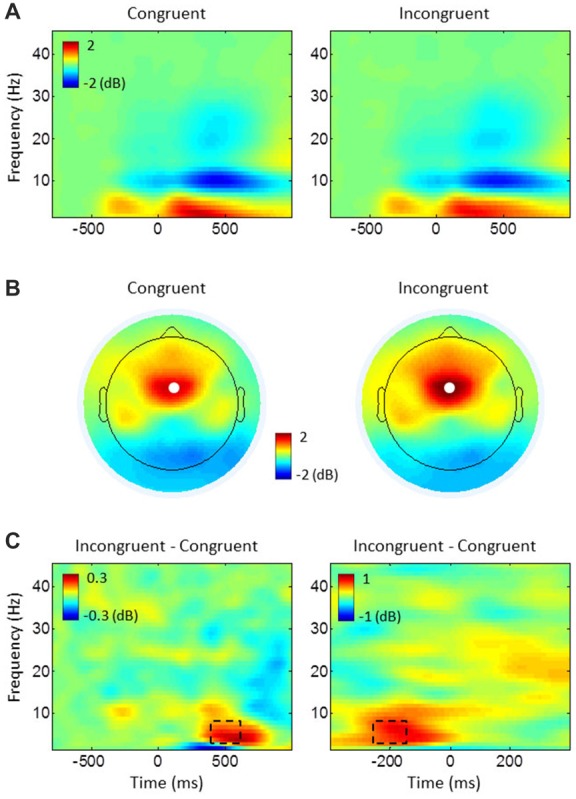
Task-related power changes relative to baseline. **(A)** Stimulus-locked power plots, averaged over all electrodes and participants for congruent and incongruent. **(B)** Topography maps of theta (4–8 Hz) power, averaged from 400 ms to 600 ms of the stimulus-locked epochs in **(A)**. White dots represent the electrode FCz. **(C)** The difference between conditions (incongruent-congruent) for stimulus-locked epochs (left) in **(A)** and response-locked epochs (right), averaged over all participants at electrode FCz. Dashed rectangles represent time-frequency windows of interest used for medial frontal cortex (MFC) theta power analyses.

Conflict modulation of MFC theta power was inspected by comparing time-frequency power plots of incongruent and congruent trials for pooled data from both groups (see Figure 3). This comparison showed the strongest theta-band (4–8 Hz) activity at electrode FCz, with a peak activity around 500 ms after target stimulus onset of stimulus-locked epochs and around −200 ms before response onset of response-locked epochs. The average power between 400 ms and 600 ms of the stimulus-locked epochs and between −250 ms and −150 ms of the response-locked epochs were subjected to statistical analyses.

**Figure 4:**
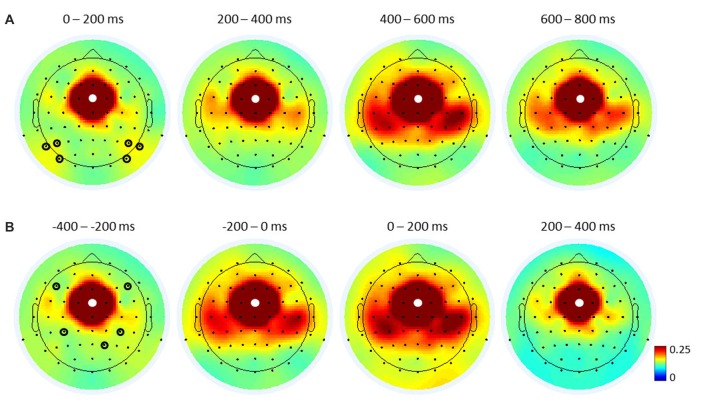
Topographical maps of FCz-seed inter-channel phase synchronization (ICPS) in theta oscillations. Stimulus-locked **(A)** and response-locked **(B)** ICPSs, averaged over all trials and participants for the respective time-windows. White dots represent the seed electrode FCz. Black circles indicate electrodes of interest used for FCz-seed ICPS analyses (see Figures 5, 7): six electrodes (P5/6, P7/8, PO7/8) in **(A)** cover the parieto-occipital cortex, and two upper electrodes (F5/6) in **(B)** cover the dorsal lateral prefrontal cortex (DLPFC), two midline electrodes (CP3/4) cover the motor cortex, and one bottom electrode (P2) covers the right parietal cortex.

**Figure 5:**
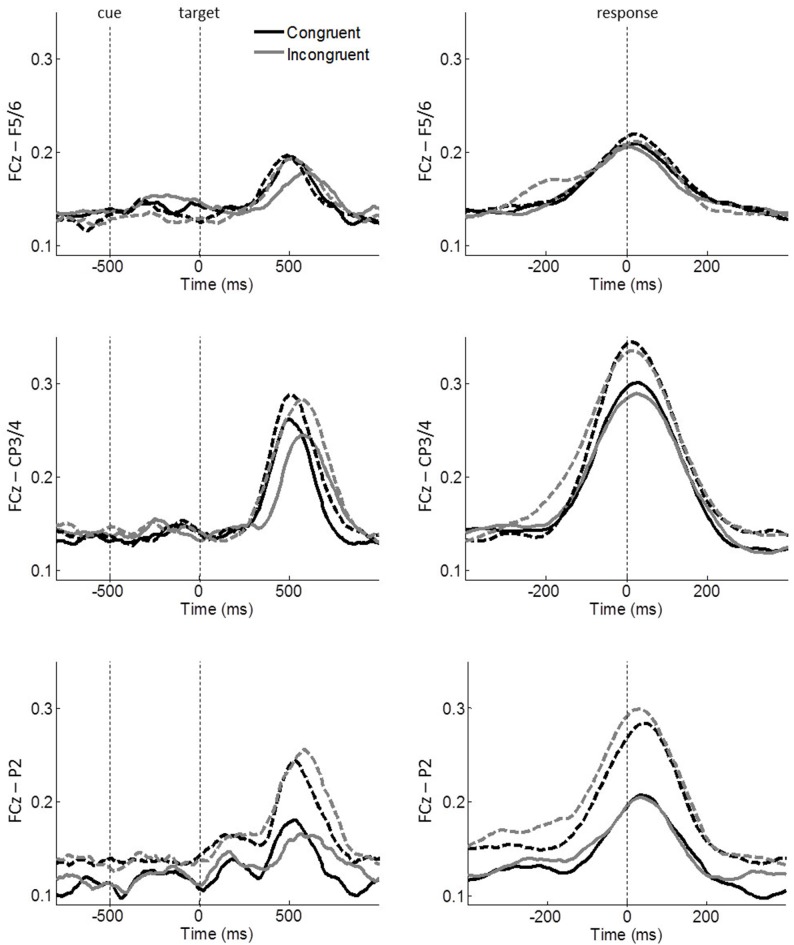
Task-related FCz-seed ICPSs in theta oscillations. Stimulus-locked and response-locked ICPSs are shown on the left and right panels, respectively. Solid and dashed lines represent controls and meditators, respectively. Black and gray lines represent congruent and incongruent trials, respectively. Averages of a 100 ms time-window around −200 ms and 0 ms of the right panels were used for ICPS analyses.

To investigate ICPSs in theta oscillations, we selected the same electrode FCz as the seed electrode. Then, regions of interest were selected based on FCz-seed ICPS topographical maps of the average of all conditions (across groups; Figure 4) and guided by the respective literature (Cavanagh et al., 2009; van de Vijver et al., 2011; Nigbur et al., 2012). We chose electrodes F5/6 to cover the DLPFC, electrodes CP3/4 for the motor cortex, and electrode P2 for the right parietal cortex. Since congruent and incongruent trials showed different distributions of RTs (see left panel in Figure 2) and ICPS activities were more closely aligned to the response onset than target stimulus onset (compare the peak activities in Figure 5), ICPS analyses were performed on response-locked epochs. The averaged ICPS activity within the same time-window as defined above (from −250 ms to −150 ms before the response onset) and a time-window showing the peak activity (from −50 ms to 50 ms around the response onset) were subjected to statistical analyses. In addition, inspection of FCz-seed ICPS topography maps led to analysis of stimulus-related effect on ICPS between the MFC and the parieto-occipital cortex (Figure 4A). Therefore, we selected further electrodes (P5/6, P7/8 and PO7/8 (PO)) to cover this brain area.

### Statistical Analyses

Effects of conflict modulation and group on RT, error rate (ER) and MFC theta power were separately tested using a 2 × 2 repeated measures analysis of variance (ANOVA) with *congruency* (congruent, incongruent) as a within-subject factor and *group* (controls, meditators) as a between-subject factor. For the analyses of MFC-centered theta network, first, the effect on ICPSs of selected electrodes was tested using a 3 × 2 × 2 repeated measures ANOVA with *electrode* (FCz–F5/6, FCz–CP3/4, FCz–P2) and *congruency* (congruent, incongruent) as within-subject factors and *group* (controls, meditators) as a between-subject factor. Second, to further specify the functional connections, each pair of electrodes was separately subjected to a 2 × 2 repeated measures ANOVA with *congruency* (congruent, incongruent) as a within-subject factor and *group* (controls, meditators) as a between-subject factor. These follow up ANOVAs are confirmative analyses of the effects found in the main ANOVA to highlight which connection shows the most prominent effect, rather than determining significant effects *per se*.

## Results

### Behavioral Results

Participants responded faster and made fewer errors during congruent trials (RT: mean = 516.798 ms, SE = 7.598; ER: mean = 1.045%, SE = 0.203) than incongruent trials (RT: mean = 588.300 ms, SE = 10.241; ER: mean = 4.322%, SE = 0.598) as reflected by significant main effects of *congruency* for both RT (*F*_(1,43)_ = 333.41, *p* < 0.001, *η*^2^ = 0.886) and ER (*F*_(1,43)_ = 41.87, *p* < 0.001, *η*^2^ = 0.493). This conflict effect showed no differences between groups in terms of RTs (the left panel in Figure 2), resulting in a non-significant *group* effect (*F*_(1,43)_ = 0.077, *p* = 0.783, *η*^2^ = 0.002) and a non-significant *congruency* × *group* interaction (*F*_(1,43)_ = 1.535, *p* = 0.222, *η*^2^ = 0.034). In contrast to RTs, fewer ERs were found among meditators (the right panel in Figure 2) as reflected by a significant main effect of *group* (*F*_(1,43)_ = 4.416, *p* = 0.041, *η*^2^ = 0.093) and *congruency* × *group* interaction (*F*_(1,43)_ = 5.263, *p* = 0.027, *η*^2^ = 0.109). *Post hoc* comparisons showed that the group difference is mainly due to incongruent trials (controls: mean ER = 5.676%, SE = 1.061; meditators: mean = 2.417%, SE = 0.515) than congruent trials (controls: mean ER = 1.238%, SE = 0.315; meditators: mean = 0.852%, SE = 0.254). Therefore, these results indicate that meditators responded more accurately than controls irrespective of RTs, especially after conflicts (see also Figure 3 in Jo et al., 2016).

### MFC Theta Power

Time-frequency power plots show increased theta power (4–8 Hz) during incongruent compared to congruent trials within 400–600 ms after target stimulus onset (stimulus-locked epochs) and between −250 ms and −150 ms before response onset (response-locked epochs; see Figures 3A,C). Spatial specificity of this theta power, assessed by topographical maps shows the strongest theta power over the electrode FCz (Figure 3B). As expected, the ANOVAs on FCz theta power revealed enhanced power in incongruent trials compared to congruent ones in stimulus-locked epochs (congruent: mean = 1.763 dB, SE = 0.203; incongruent: mean = 2.185 dB, SE = 0.218; *F*_(1,43)_ = 14.192, *p* < 0.001, *η*^2^ = 0.248) and in response-locked epochs (congruent: mean = 1.098 dB, SE = 0.173; incongruent: mean = 1.757 dB, SE = 0.188; *F*_(1,43)_ = 56.841, *p* < 0.001, *η*^2^ = 0.569). Although meditators showed higher power in congruent (mean difference between groups in stimulus-locked epochs = 0.276 dB, response-locked epochs = 0.227 dB) and incongruent trials (stimulus-locked epochs = 0.199 dB, response-locked epochs = 0.056 dB), we found no other significant main effects or interactions indicating group difference for both stimulus- and response-locked epochs (all *p* ≥ 0.352, all *η*^2^ ≤ 0.020).

### MFC–Centered Theta Network

The *electrode* × *congruency* × *group* ANOVA on ICPS before response onset (average between −250 ms and −150 ms of response-locked epochs) revealed a significant main effect of *congruency* (*F*_(1,43)_ = 10.639, *p* = 0.002, *η*^2^ = 0.198), showing an overall enhanced synchrony during incongruent (mean = 0.158, SE = 0.008) compared to congruent trials (mean = 0.144, SE = 0.006). Furthermore, we found a significant interaction of *congruency* × *group* (*F*_(1,43)_ = 5.049, *p* = 0.030, *η*^2^ = 0.105). No other main effects or interactions were significant (all *p* ≥ 0.185, all *η*^2^ ≤ 0.041). Additionally, lateralization effect was also examined by comparing ICPSs between FCz–F5 and FCz–F6 and between FCz–CP3 and FCz–CP4 for each congruent and incongruent condition and the results showed no significant effects (two-tailed paired *t*-test, all *p* ≥ 0.492).

To further specify the significant effects, follow-up *congruency* × *group* ANOVAs were performed on each pair of electrodes, i.e., FCz–F5/6, FCz–CP3/4, FCz–P2. A significant main effect of *congruency* was observed on FCz–CP3/4 (*F*_(1,43)_ = 10.263, *p* = 0.003, *η*^2^ = 0.193) and FCz–P2 (*F*_(1,43)_ = 5.199, *p* = 0.028, *η*^2^ = 0.108; Figure 5), while FCz–F5/6 did not reach significance (*F*_(1,43)_ = 2.244, *p* = 0.141, *η*^2^ = 0.050). The *congruency* × *group* effect was observed on FCz–CP3/4 (*F*_(1,43)_ = 5.230, *p* = 0.027, *η*^2^ = 0.108), indicating that ICPS is comparable between groups during congruent trials (controls: mean = 0.147, SE = 0.10; meditators: mean = 0.143, SE = 0.010) but meditators exhibited considerable enhanced synchrony compared to controls during incongruent trials (controls: mean = 0.152, SE = 0.012; meditators: mean = 0.174, SE = 0.013). No other main effects or interactions were significant (all *p* ≥ 0.102, all *η*^2^ ≤ 0.061). These results indicate conflict modulations by FCz–CP3/4 and FCz-P2 synchronies. Furthermore, meditators exhibited an increased FCz–CP3/4 synchrony after conflicts, as compared to controls.

The same analysis was applied for ICPS around response onset (average between −50 ms and 50 ms of response-locked epochs). The *electrode* × *congruency* × *group* ANOVA revealed a significant main effect of *electrode* (*F*_(2,86)_ = 14.940, *p* < 0.001, *η*^2^ = 0.258), indicating the highest synchrony between FCz and CP3/4 (mean = 0.304, SE = 0.023) followed by FCz–P2 (mean = 0.231, SE = 0.020) and FCz–F5/6 (mean = 0.205, SE = 0.018). No other main effects or interactions were significant (all *p* ≥ 0.129, all *η*^2^ ≤ 0.046).

**Figure 6:**
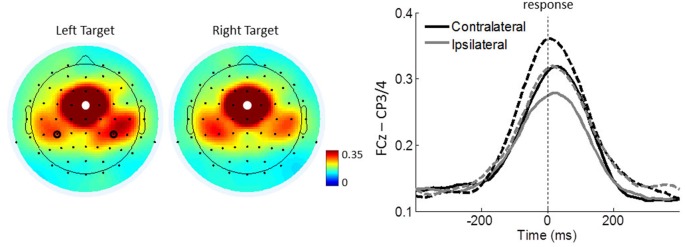
Motor-related ICPSs between FCz and CP3/4 in theta oscillations. Left panel shows the topographical maps average of 100 ms around the response onset for the right and left target trials. White dots represent the seed electrode FCz and black circles indicate electrodes CP3/4 over the motor cortex. Right panel shows FCz-seed contralateral and ipsilateral ICPSs to the responding hand. Solid and dashed lines represent controls and meditators, respectively.

To test whether the strongest ICPS over the motor cortex (FCz–CP3/4) is dependent on the responding hand, we further conducted a repeated measures ANOVA with *congruency* (congruent, incongruent) and *lateralization* (contralateral, ipsilateral) as within-subject factors and *group* (controls, meditators) as a between-subject factor. It revealed a significant main effect of *lateralization* (*F*_(1,43)_ = 21.470, *p* < 0.001, *η*^2^ = 0.333), indicating that ICPS between FCz and CP3/4 is enhanced on contralateral (mean = 0.323, SE = 0.025) compared to ipsilateral areas (mean = 0.281 SE = 0.022; Figure 6). No other main effects or interactions were significant (all *p* ≥ 0.386, all *η*^2^ ≤ 0.018). These results indicate that FCz–CP3/4 synchrony around response onset is specific to the responding hand.

**Figure 7:**
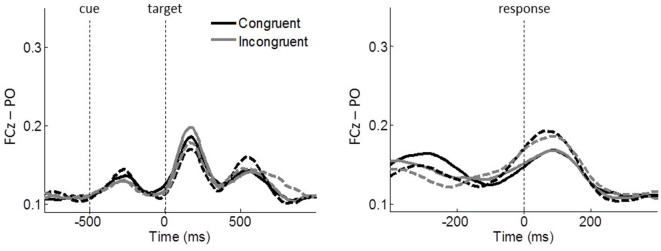
Task-related ICPSs between FCz and PO (P5/6, P7/8, PO7/8) in theta oscillations. Stimulus-locked and response-locked ICPSs are shown on the left and right panels, respectively. Solid and dashed lines represent controls and meditators, respectively. Black and gray lines represent congruent and incongruent trials, respectively.

Lastly, we quantified the stimulus-related effect on ICPS between FCz and PO. In contrast to the other theta phase synchronizations, FCz–PO is more closely aligned to the stimulus onset than to the response onset (Figure 7), which shows maximal activity around 180 ms after the onset of the target stimulus in stimulus-locked epochs and around 80 ms after response in response-locked epochs. The *congruency* × *group* ANOVA around these peak activities (average within 130–230 ms and 30–130 ms of stimulus- and response-locked epochs, respectively), revealed no significant main effects or interactions (all *p* ≥ 0.094, all *η*^2^ ≤ 0.064), indicating the absence of conflict effects or group differences.

### Correlation Analysis

**Figure 8:**
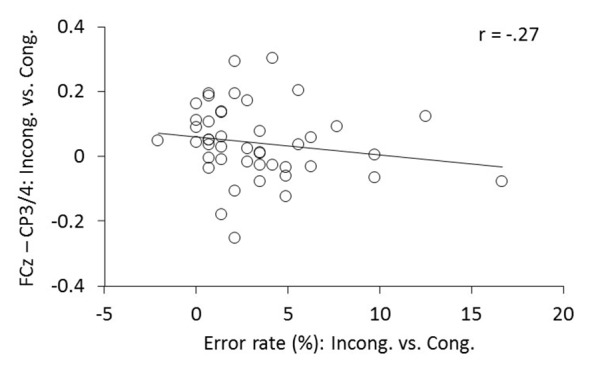
The relationship between the changes in FCz-CP3/4 synchrony and ER. Individual changes of ICPSs (ln[incongruent] − ln[congruent]) shows corresponding changes in ERs (incongruent − congruent).

Having found increased ICPS between FCz and CP3/4 and fewer ERs in meditators, we further examined whether the increased theta phase synchrony over the motor cortex was predictive of low ER. Indeed, correlation analysis revealed that, in comparison to congruent trials, individuals with higher increase of ICPS between FCz and CP3/4 after conflict generally showed lower ER (Spearman’s coefficient *r* = –0.271; *p* = 0.036, one-tailed; Figure 8).

## Discussion

The present study investigated frontal theta dynamics of long-term mindfulness meditators in comparison to non-meditating age- and gender-matched controls using the flanker-type ANT. Meditators made significantly fewer errors than controls in conflict trials, while RTs were comparable between groups. In the presence of this behavioral group difference, we examined whether meditators show different neural activity of MFC theta power and/or MFC-centered theta network compared to controls. Mindfulness meditators showed enhanced MFC-centered theta network after conflicts, while no group effect was found for MFC theta power.

In line with previous findings of conflict effect, the flanker-type ANT effectively replicated the influence of response conflict on behavioral performances and theta oscillations over the MFC, as well as their phase synchrony with other brain areas (for a review see Cavanagh and Frank, 2014). Previous research demonstrated MFC theta dynamics for spatial conflicts (Cohen and Ridderinkhof, 2013), semantic conflicts (Jiang et al., 2015), as well as for conflicts on stimulus and response levels (Nigbur et al., 2012). Furthermore, the evidence that error-related processing after conflicts evokes MFC theta dynamics (Cavanagh et al., 2009; van Driel et al., 2012) accounts for the engagement of cognitive control relevant to the detection and correction of errors. In accordance with the literature, the present study showed that after conflicts both meditators and controls exhibited increased MFC theta power and functional connections on MFC-centered theta network, with increased response times and ERs.

A closer look at the MFC-centered theta network revealed that the respective conflict modulation of the network is more closely aligned to the time of the response onset than to the cue or target stimulus onset (Figure 5). The effect of a response-related lateralization over the motor cortex (Figure 6) strengthens the argument for the role of specific functional linkages, rather than a mere effect of re-alignment in time (i.e., response-locked epochs). Additionally, the lack of similar response-locked functional connectivity between the MFC and the parieto-occipital cortex also supports this argument (Figure 7). On the other hand, the early functional connection of FCz–PO is more closely aligned to the stimulus onset, thus reflecting stimulus-related effects. These findings are in line with the notion that the MFC-centered theta network is specifically involved in response controlling functions (Nigbur et al., 2012). Increased phase synchrony restricted to theta-band oscillations between the motor cortex and muscle to the responding hand (van de Vijver et al., 2011) further support the functional role of theta phase synchrony during action adjustment.

In a previous study, an enhanced functional connectivity between the MFC and DLPFC was observed after conflicts. However, in the current study, the FCz–F5/6 synchrony was not significantly modulated. Previous studies on this functional connection have compared error responses with correct responses (Cavanagh et al., 2009; van de Vijver et al., 2011; van Driel et al., 2012) and response conflicts with stimulus conflict trials (Nigbur et al., 2012). These conditions emphasize error-related, as well as response conflict effects, and thus could provide a distinct MFC-centered theta network. However, the current study included only correct response trials, as the ANT paradigm modulated response conflicts and showed generic lower ER. Furthermore, although the ANT paradigm has been shown to assess executive conflict function independently from cueing conditions (Fan et al., 2002), we cannot rule out a possible effect of cue-induced expectation on MFC-centered theta network. The cue stimulus, which facilitates responding by either alerting or orienting to the forthcoming target stimulus, may reduce the resource requirement for the target anticipation during response conflict processing (Fan et al., 2007). Nonetheless, it is noteworthy that, in agreement with previous studies, a trend of enhanced FCz–F5/6 synchrony during incongruent trials was observed shortly before the response onset among meditators (congruent: mean = 0.147, SE = 0.014; incongruent: mean = 0.167, SE = 0.017; two-tailed paired *t*-test, *p* = 0.082; see the upper right panel in Figure 5).

Compared to the control group, mindfulness meditators showed similar RT and MFC theta power. However, they made fewer errors and expressed an enhanced functional connection about 200 ms before the response onset, in particular between the MFC and the motor cortex during incongruent trials. Thus, on the behavioral level, irrespective of response timing, the accuracy after conflicts was significantly better in meditators than in controls. The higher amplitude of the parietal P3 component has provided neural evidence for this behavioral efficiency in meditators (see Figure 6 in Jo et al., 2016). P3 amplitude was closely aligned to target stimulus onset, suggesting increased attentional engagement to an attended central target. On the other hand, in the current study with the same participants (Jo et al., 2016), we found enhanced synchrony in theta oscillations between the MFC and motor cortex. Notably, this connection is more closely aligned to the response onset, suggesting its direct involvement in response controlling function (van de Vijver et al., 2011; Nigbur et al., 2012). Therefore, the pattern of results from the current study combined with previous findings (Jo et al., 2016) suggest that mindfulness meditators might efficiently allocate their attentional resources to the target stimulus making input selection more focused, and also increase their control of response conflicts over the motor system in association with the MFC-centered theta network. A negative correlation of individual changes in FCz-CP3/4 theta phase synchrony (incongruent vs. congruent), with the corresponding changes in ERs, further implicates the functional role of the MFC-centered theta network for action control. Further research on individuals with cognitive impairments such as schizophrenia, attention deficit hyperactivity disorder (ADHD) and multiple sclerosis would be of interest with respect to the association with the MFC-centered theta network. These populations exhibit abnormal behavioral performance as well as conflict modulations of P3 activity (Neuhaus et al., 2007, 2010; Kratz et al., 2011; Vázquez-Marrufo et al., 2014; Hasler et al., 2016) and emerging evidence indicates that mindfulness-based approaches may be effective for example in reducing ADHD symptoms (Cairncross and Miller, 2016).

In contrast to differences in the MFC-centered theta network, the current results did not reveal a significant group difference in MFC theta power. Further analysis on baseline power (average of all trials between −300 and −100 before the cue stimulus onset) also showed no group effect (*p* = 0.586). Thus, it seems unlikely that the group difference in response accuracy is specific to MFC theta power. While the mental state of meditation may exhibit higher levels of theta power (Lomas et al., 2015; Brandmeyer and Delorme, 2016) and the present study found no group effect on baseline theta power, the increased theta phase synchrony in meditators may not be due to their meditation state. Rather it is suggested that meditators might engage in adjusting their behavior after the target presentation, as reflected by the response-aligned MFC-centered theta network. Previous research has indicated that, while MFC theta power is associated with detecting the presence of conflict, the MFC-centered theta network is more relevant to the engagement of task-specific processing to avoid errors (see “Introduction” Section). However, as MFC theta power is obviously related to the MFC-centered theta network, it is not possible to entirely rule out the impact of MFC theta power on behavioral performance. Further studies with individuals who demonstrate different RTs with comparable ERs would help to reveal the functional roles by which MFC theta dynamics improves conflict detection and the timing of the motor onset.

To conclude, mindfulness meditators showed efficient cognitive control after conflicts as reflected by fewer error responses irrespective of response timing. Furthermore, meditators showed enhanced conflict modulations of functional connection between the MFC and the motor cortex, while MFC theta power showed no difference compared to the control group. This pattern of results suggests that the selective enhancement of frontal theta dynamics might render efficient cognitive control over the motor system. Our study is consistent with the idea that the MFC-centered network is relevant to the involvement in resolving conflicts to avoid errors.

## Author Contributions

SS designed and conducted the experiment. H-GJ analyzed the data. H-GJ, PM and SS interpreted the data and wrote the article.

## Conflict of Interest Statement

The authors declare that the research was conducted in the absence of any commercial or financial relationships that could be construed as a potential conflict of interest.
